# A significant number of pediatric inflammatory bowel disease patients are exposed to a medication not approved by the Food and Drug Administration for pediatric use

**DOI:** 10.1002/jpn3.70200

**Published:** 2025-08-25

**Authors:** Courtney Rusch, Anthony J. Perkins, Steven J. Steiner, the ImproveCareNow Pediatric IBD Learning Health System

**Affiliations:** ^1^ Division of Pediatric Gastroenterology, Hepatology and Nutrition, Riley Hospital for Children Indiana University School of Medicine Indianapolis Indiana USA; ^2^ Department of Biostatistics and Health Data Science Indiana University School of Medicine Indianapolis Indiana USA

**Keywords:** biologic therapy, Crohn's disease, ulcerative colitis

## Abstract

**Objectives:**

Inflammatory bowel disease (IBD) presents before age 20 years in ~25% of patients. Regulatory approvals of IBD medications for pediatric use are often delayed. Nevertheless, many pediatric patients receive medication not approved for pediatric use. The aim of this study was to summarize the exposure of pediatric patients to IBD medication without regulatory approval for pediatrics.

**Methods:**

A retrospective study of exposure to nonapproved biologic/small molecule medications in patients listed in the ImproveCareNow registry with IBD diagnosed before September 2023 was conducted. Chi‐square tests were used to see whether exposure differed by demographics, the cohort of IBD diagnosis (Crohn's disease [CD], ulcerative colitis [UC]), or exposure timeframe (i.e., age at exposure and time from diagnosis to exposure).

**Results:**

16,085 eligible patients with year of diagnosis from 1993 to 2023 were identified. 2836 patients (17.6%) were exposed to a medication not approved by the Food and Drug Administration (FDA) for pediatric use. The mean age of exposure was 12.5 years and the mean time from diagnosis to exposure was 2.9 years. Patients with UC (23.8%) were significantly more likely to have exposure than CD (15.3%). Female patients (19.1%) were more likely to have exposure than male patients (16.5%). Patients diagnosed with IBD between ages 0–5 years had the highest rate of exposure and shortest time from diagnosis to exposure.

**Conclusions:**

A significant number of pediatric IBD patients were exposed to medication not approved by the FDA for pediatrics. There is an urgent need for more rapid approval of medications for IBD in pediatrics.

## Abstract (highlights)

**What is Known:**

- Significant delays exist in obtaining pediatric approval for inflammatory bowel disease (IBD) therapies.
- A large number of pediatric IBD patients are exposed the therapies which are not approved for pediatric use.

**What is New:**

- Using the ImproveCareNow pediatric registry, a survey of >16,000 pediatric IBD patients was conducted, revealing that >17% were exposed to a medication not approved for pediatric use.
- Risk factors for use of nonapproved medications included diagnosis of ulcerative colitis and female sex.

## Graphical abstract

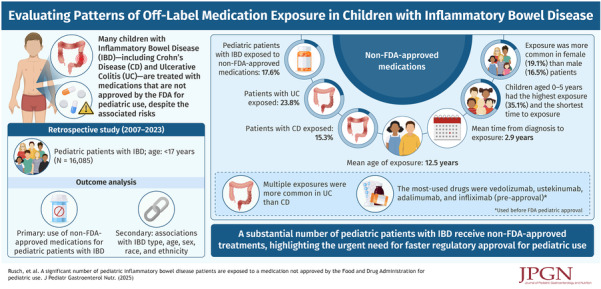

## 1 INTRODUCTION

Inflammatory bowel disease (IBD) is a chronic, autoimmune disease characterized by inflammation in the gastrointestinal tract. Specific features of disease further categorize IBD into Crohn's disease (CD), ulcerative colitis (UC), and IBD‐Unspecified (IBD‐U) or “indeterminate colitis.” It is estimated that 25% of patients present with IBD before 20 years of age.^1^ Approximately 18% of children with IBD are diagnosed before the age of 10, and 4% before the age of 5.^2^ In the United States, the prevalence of IBD is 100–200 per 100,000 children. Presentation of IBD is variable, but patients typically present with a constellation of symptoms that can include abdominal pain, weight loss, diarrhea, rectal bleeding, and extraintestinal manifestations.^3^ After diagnosis, the goals of therapy include clinical symptom improvement, mucosal healing, growth restoration, returning to a normal quality of life, minimizing adverse effects of medications, and eliminating complications. Potential long‐term complications of under‐treated disease include micronutrient deficiencies, linear growth impairment, abnormal bone metabolism, and colon cancer.^4^, ^5^, ^6^, ^7^, ^8^

Therapeutic options for IBD are carefully selected to induce remission of active disease, and then to maintain clinical and mucosal remission once achieved. The selected therapy or therapies depends primarily on the severity of active disease, the patient's tolerance, observed clinical response, and cost. Options include corticosteroids, enteral nutrition therapy, aminosalicylates, immunomodulators, biologics, targeted synthetic small molecules, and surgery.^1^, ^9^, ^10^ Timely access to selected medications is critical in treatment of IBD and can potentially prevent complications from untreated disease such as strictures, infection, or need for surgical intervention.^11^, ^12^, ^13^, ^14^ United States Food and Drug Administration (FDA) approvals of medications for pediatric use often have long delays after adult approval for IBD.^15^ It is common for pediatric patients prescribed biologics/small molecules to experience delays in receiving the medication.^16^ These delays put patients at risk for complications from their disease, but utilization of non‐FDA‐approved IBD medications in children can be lifesaving and improve quality of life significantly when their disease is in remission.^17^, ^18^ Of the approximately 13 approved biologic/small molecule products currently available for adults, the only therapies that were approved in children at the time of analysis are infliximab (IFX) and adalimumab, both of which are antitumor necrosis factor agents.

Infliximab (IFX) was the first biopharmaceutical with FDA approval for adult CD in 1998 and adult UC in 2005. Subsequent approval for pediatric CD ages 6 years and older occurred in 2006 and pediatric UC ages 6 years and older in 2011.^19^ Adalimumab was FDA approved for adult CD in 2007 and adult UC in 2012. Pediatric approval of adalimumab for CD ages 6 years and older occurred in 2014 with approval for UC ages 5 years and older in 2021.^20^ At the time of analysis, there were no other FDA‐approved biologic or small molecule therapies for pediatric IBD. The goal of this study was to review non‐FDA‐approved medications used in the routine care of pediatric IBD patients. The primary aim was to observe the incidence of pediatric patients with IBD that have been exposed to an adult‐approved biologic or small molecule without pediatric FDA approval.

## 2 METHODS

This is a retrospective study of patients enrolled in the ImproveCareNow (ICN) registry with IBD (CD, UC, or IBD‐U). ICN is an international multicenter registry that enrolls pediatric IBD patients receiving their care at participating centers, with the goal of advancing pediatric IBD care through quality improvement and research. Only records in existence at the time of IRB review and approval were accessed for review in this study. Inclusion criteria for this study included all patients enrolled in the ICN Registry from 2007 to September 2023 who were less than 17 years of age at the time of their IBD diagnosis with at least 1 year of follow‐up using ICN's comprehensive clinical data. A patient's data were only utilized if they were started on a medication before official FDA approval for their age. Only patients with a diagnosis of CD or UC were included for analysis. All included patients were treated at an institution within the United States. Exclusion criteria included patients with an IBD diagnosis of IBD‐U. Patients without at least 1 year of follow‐up documented in the ICN registry were excluded from the study.

The primary outcome variable in this study is observation of non‐FDA‐approved medications used in routine care of pediatric patients with IBD. The medications being analyzed for their use in pediatrics have FDA approval for the adult IBD population. The hypothesis is that nonpediatric FDA‐approved biologics/small molecule medications are routinely used for treatment of pediatric patients with IBD. Secondary outcomes include identifying which factors, such as IBD phenotype, age, race, ethnicity, or gender, contribute to increased use of medications not approved for pediatric IBD patients.

Patients were stratified into prespecified subgroups including by gender and by cohort of IBD diagnosis (CD and UC). Additional variables considered in analysis of this data set included: patient age, date of diagnosis, gender, race, ethnicity, biologic, and small molecule use. The primary list of medications observed included IFX, adalimumab, vedolizumab, ustekinumab, ozanimod, upadacitinib, risankizumab, natalizumab, certolizumab, tofacitinib, and all biosimilars within this list. Each of the aforementioned medications have FDA approval in adult IBD.

Chi‐square tests were conducted to determine if the percentage of patients receiving a biologic without FDA approval differs by specific groups of interest (gender, IBD diagnosis, etc.). Kaplan–Meier plots and log‐rank tests were utilized to examine if the time to receipt of a biologic without FDA approval differed by gender and IBD diagnosis. All analyses were conducted using SAS v9.4. A *p*‐value of <0.05 was considered statistically significant. All variables are presented as mean (±standard deviation) or *n* (%).

### 2.1 Ethics statement

This study was approved by the Institutional Review Board of Indiana University and by the ICN Research Committee. All patients within the ICN registry in this study provided informed consent for use of their data for research.

## 3 RESULTS

A total of 16,085 eligible patients were identified within the ICN registry and were assessed for their exposure to a medication not approved by the FDA for pediatric use. The mean age at diagnosis was 11.1 (±3.6) years. 56.2% were male and 83.4% were white. Patients were first differentiated by IBD diagnosis and subsequently divided by disease phenotype. 11,613 patients (72.2%) had CD, and most of these patients had inflammatory, nonpenetrating, and nonstricturing disease (82.5%). Of the 4472 patients (27.8%) with UC, 64.8% had pancolitis. Baseline characteristics of this cohort can be found in Table 1.

**Table 1:** Baseline characteristics of subjects.

Patient demographics, *n* = 16,085	
Mean age at diagnosis (SD)	11.1 (3.6)
Gender, *n* (%)	
Male	9043 (56.2)
Female	7042 (43.8)
Race, *n* (%)	
American Indian or Alaska Native	40 (0.3)
Asian	406 (2.8)
Black	1826 (12.6)
Multiracial	101 (0.7)
Native Hawaiian or other Pacific islander	29 (0.2)
White	12,087 (83.4)
Diagnosis, *n* (%)	
Crohn's disease	11,613 (72.2)
Ulcerative colitis	4472 (27.8)
Crohn's disease phenotype at enrollment, *n* (%)	
Inflammatory, nonpenetrating, nonstricturing	8426 (82.5)
Stricturing only	788 (7.7)
Penetrating only	723 (7.1)
Both stricturing and penetrating	272 (2.7)
Extent of ulcerative colitis disease ate enrollment, *n* (%)	
Ulcerative proctitis (rectum only)	335 (8.4)
Left side ulcerative colitis (distal to the splenic fixture only)	671 (16.8)
Extensive ulcerative colitis (extend proximal to the splenic flexure)	358 (8.9)
Pancolitis (the entire colon)	2598 (64.8)
Not assessed completely (colonoscopy incomplete)	44 (1.1)

2836 patients (17.6%) were exposed to a medication not approved by the FDA for pediatric use. Patients with UC were significantly more likely to receive medications lacking FDA approval in pediatrics compared to those with CD (Figure S1). Specifically, the exposure rates were 23.8% for UC patients and 15.3% for those with CD (*p* < 0.001). Further analysis revealed that there were no significant differences in the exposure rates among the various phenotypes of CD. However, within the UC cohort, patients presenting with pancolitis exhibited a notably higher exposure rate of 26% (*p* < 0.001) than other UC phenotypes (Table 2).

**Table 2:** Percentage of patients taking medication without Food and Drug Administration pediatric approval by gender, race, year of diagnosis, and disease (*n* = 16,085).

	# of patients taking medication without FDA pediatric approval, *n* (%)	*p* value
Gender		<0.001
Male (*n* = 9043)	1490 (16.5)	
Female (*n* = 7042)	1346 (19.1)	
Race		0.706
American Indian/Alaskan Native (*n* = 40)	10 (25.0)	
Asian (*n* = 406)	74 (18.2)	
Black (*n* = 1826)	328 (18.0)	
More than one race (*n* = 101)	20 (19.8)	
Native Hawaiian/Other Pacific Islander (*n* = 29)	7 (24.1)	
White (*n* = 12,087)	2120 (17.5)	
Year of diagnosis		<0.001
2000 or earlier (*n* = 111)	2 (1.8)	
2001–2005 (*n* = 671)	46 (6.9)	
2006–2010 (*n* = 2124)	280 (13.2)	
2011–2015 (*n* = 5010)	839 (16.8)	
2016–2020 (*n* = 6414)	1365 (21.3)	
2021–2023 (*n* = 1342)	215 (16.0)	
Crohn's disease phenotype		0.929
Inflammatory, nonpenetrating, nonstricturing (*n* = 8426)	1315 (15.6)	
Stricturing only (*n* = 788)	117 (14.8)	
Penetrating only (*n* = 723)	116 (16.0)	
Both stricturing and penetrating (*n* = 272)	43 (15.8)	
Ulcerative colitis extent of disease at enrollment		<0.001
Ulcerative proctitis (*n* = 335)	52 (15.5)	
Left side ulcerative colitis (*n* = 671)	144 (21.5)	
Extensive ulcerative colitis (*n* = 358)	79 (22.1)	
Pancolitis *(n* = 2598)	676 (26.0)	
Not assessed completely (*n* = 44)	8 (18.2)	
Age at diagnosis		<0.001
0–5 (*n* = 1452)	509 (35.1)	
6–9 (*n* = 3392)	724 (21.3)	
10–13 (*n* = 6474)	1066 (16.5)	
14–17 (*n* = 4767)	537 (11.3)	

The mean age at which patients first encountered medications without pediatric FDA approval was 12.5 (±3.9) years and the average time from diagnosis to exposure was approximately 2.9 (±2.6) years (Table 3). Although patients diagnosed with IBD at a very young age, less than 6 years of age, represented the smallest cohort in this study (Table 2), they experienced a shorter duration from diagnosis to exposure compared to other age ranges (Figure S2). Additionally, they had the highest rate of exposure to a non‐FDA‐approved medication of 35.1% (*p* < 0.001). Among pediatric patients 6 years and older, the older pediatric patients (ages 14–17 years) had the shortest time to exposure (Figure S2).

**Table 3:** Patients exposed to any medication without Food and Drug Administration pediatric approval (*n* = 2836).

Time to exposure, *n* (%)	
Within 1 year of diagnosis	776 (27.4)
1–2 years from diagnosis	615 (21.7)
2–5 years from diagnosis	919 (32.4)
More than 5 years from diagnosis	526 (18.5)
Mean time (Years) from diagnosis to exposure (SD)	2.9 (2.6)
Age at exposure in years, *n* (%)	
<6	295 (10.4)
6–9	274 (9.7)
10–13	851 (30.0)
14–17	1416 (49.9)
Mean age at exposure (SD)	12.5 (3.9)

Abbreviation: SD, standard deviation.

This analysis revealed gender disparities in exposure rates, with female patients (19.1%) being more likely to receive non‐FDA‐approved medications than male patients (16.5%) (*p* < 0.001). Gender disparities were noted in the specific diagnosis categories: 41% of Crohn's patients were female while 51% of UC patients were female (*p* < 0.001). Among the CD patients, females had a higher rate of exposure (17.0%) than males (14.1%), whereas exposure was quite similar in the UC patients with 23.6% of females and 23.9% of males having exposure. In both genders, UC patients had higher rates of exposure than CD. In females, 23.6% of UC patients were exposed compared to 17.0% of CD patients. In males, 23.9% of UC patients were exposed compared to 14.1% of CD patients. No significant differences were observed in medication exposure when stratified by race or ethnic groups.

A bivariate analysis was performed to look at any non‐FDA‐approved medication exposure within the CD and UC cohorts (Table 4). Exposure to adalimumab and its biosimilars (*n* = 894) before their approval for pediatric IBD was more frequent in UC patients (*p* < 0.001). Similarly, exposure to IFX and its biosimilars (*n* = 240) before approval for pediatric IBD was more frequent in UC patients (*p* < 0.01). CD patients were more frequently exposed to certolizumab (*n* = 63) and Ustekinumab (*n* = 1138) (*p* < 0.001). UC patients were more frequently exposed to vedolizumab (*n* = 1057), golimumab (*n* = 23), and tofacitinib (*n* = 69) (*p* < 0.001). There was no significant difference in exposure for natalizumab (*n* = 3).

**Table 4:** Number of patients exposed to specific medications^a^ without FDA pediatric approval.

	Total patients *n* = 16,085	Crohn's disease n = 11,613	Ulcerative colitis *n* = 4472	*p* value
Adalimumab	894 (5.5)	424 (3.6)	470 (10.5)	<0.001
Infliximab	240 (1.5)	153 (1.3)	87 (1.9)	0.005
Certolizumab	63 (0.4)	59 (0.5)	4 (0.1)	<0.001
Vedolizumab	1057 (6.6)	527 (4.5)	530 (11.8)	<0.001
Golimumab	23 (0.1)	8 (0.1)	15 (0.3)	<0.001
Ustekinumab	1138 (7.1)	959 (8.3)	179 (4.0)	<0.001
Tofacitinib	69 (0.4)	18 (0.2)	51 (1.1)	<0.001
Natalizumab	3 (0.02)	3 (0.03)	0 (0.0)	0.565
Any medication	2836 (17.6)	1773 (15.3)	1063 (23.8)	<0.001
Number of medications				<0.001
0	13,249 (82.4)	9840 (84.7)	3409 (76.2)	
1	2273 (14.1)	1442 (12.4)	831 (18.6)	
2	484 (3.0)	290 (2.5)	194 (4.3)	
3	67 (0.4)	32 (0.3)	35 (0.8)	
4	9 (0.06)	6 (0.05)	3 (0.07)	
5	3 (0.02)	3 (0.03)	0 (0.0)	

*Note*: Percentage of each cohort expressed in parentheses. *p* value is comparison between frequencies in the Crohn's disease and ulcerative colitis cohorts.

Abbreviation: FDA, Food and Drug Administration.

^a^ Numbers presented for adalimumab and infliximab are before FDA approval for the relative pediatric indications.

As additional medications were approved for adult IBD during the existence of the ICN registry, frequency of exposure increased concomitantly in pediatric patients. Rates of exposure in pediatric patients increased when analyzed by year of diagnosis of pediatric patients from 2000 and earlier (1.8%) through diagnosis year of 2016 through 2020 (21.3%) (Table 2). Pediatric patients diagnosed in 2021 through 2023 had slightly lower rates of exposure (16.0%) than patients diagnosed between 2016 and 2020 (21.3%), perhaps due to shorter time from diagnosis at the time of analysis. A more detailed analysis by year of diagnosis from 2007 through 2022 revealed a gradual increase in exposure to nonapproved medications (Table S1), notably from year of diagnosis 2007 (12.2%) through the year of diagnosis with highest exposure rate, 2018 (22.5%).

Rates of exposure to non‐FDA‐approved medications increase as time from diagnosis increases, likely reflecting use of approved medications initially (since the approval by the FDA of both IFX and adalimumab for pediatric use), with change to non‐FDA‐approved medications with initial medication failure (i.e., nonresponse, loss of response, or adverse event). When all patients were examined for time from diagnosis to first exposure to a non‐FDA approved medication use, a substantial percentage of patients undergo this exposure within 1 year of diagnosis (27.4%), while first exposure continues between 1 and 2 years from diagnosis (21.7%), between 2 and 5 years from diagnosis (32.4%), and 5 years and longer from diagnosis (18.5%) (Table 3).

In addition to a higher frequency of single medication exposures, UC patients were exposed to a greater total number of non‐FDA‐approved medications for pediatric IBD (*p* < 0.001). More than 5% of UC patients were exposed to 2 or more medications without pediatric approval, compared to almost 3% of CD patients (Table 4).

## 4 DISCUSSION

This study provides critical insights into pediatric IBD patient exposure to medications not approved by the FDA for pediatric use. Our findings indicate that a substantial proportion of children with IBD (17.6%) were exposed to such medications. These data highlight a significant gap in the approved therapeutic options available to this vulnerable population. In fact, there has been a long history of the use of medications not approved by the FDA for IBD, including some forms of corticosteroids and various immunomodulators, such as methotrexate and thiopurines.^21^, ^22^

Medication exposure rates varied when demographic differences were analyzed. This raises important questions regarding factors that may influence the need for advanced medications in pediatric IBD. Our results showed that patients with UC, particularly those with pancolitis, were more likely to be exposed to non‐FDA‐approved treatments compared to those with CD. This disparity could reflect more severe disease presentations within the UC cohort at baseline and during disease course. It might also reflect evolving treatment strategies, that is, “treat to target,” as specified in the STRIDE‐II initiative.^23^ Physicians may choose to utilize non‐FDA‐approved medications sooner in more severe cases to prevent complications of undertreated IBD. Another consideration for this observation is that both IFX and adalimumab had later FDA approval years for the indication of pediatric UC versus pediatric CD. Thus, its prevalence in our data may be reflective of the off‐label use and not necessarily baseline disease severity.

Our data reveal that female patients had a higher likelihood of receiving non‐approved medications than male patients, particularly in CD. This suggests that there may be gender‐based differences in disease presentation and treatment responsiveness. Further research would be warranted to explore the underlying mechanisms for this discrepancy.

Younger patients diagnosed between the ages of 0–5 had the highest rates of exposure before age 18 years to a medication not approved by the FDA for pediatric use. This may be attributed to the fact that they are followed in the pediatric setting for a longer period compared to adolescent patients that transition to adult care sooner. Interestingly, patients in this age range also had the shortest time from diagnosis to non‐FDA‐approved medication exposure. This may suggest that younger patients generally have a more severe disease state at presentation necessitating biologic or small molecule therapy sooner than children diagnosed at an older age. Our data are not sufficient to quantify the difference in disease severity and/or refractory characteristics between cohorts. This study did not incorporate laboratory results, histologic findings, or clinical reasoning from physicians, leading to the use of non‐FDA‐approved medications that may help delineate what leads to this shorter exposure timeline. The higher exposure rates may simply be secondary to the relatively longer length of time being observed before turning 18 years old. However, it is important to consider that efficient access to medications for IBD in younger age groups could mitigate complications related to pre‐pubertal development, growth, and natural disease progression that may result in surgical intervention or other long‐term complications.

The implications of our findings extend to the broader context of drug approval processes for the pediatric IBD population. The small number of available therapies approved for children, despite numerous adult options, poses significant challenges in clinical practice. The lack of FDA approval for pediatric IBD medications places these patients at a disadvantage. The delays caused by awaiting insurance approval for use of a non‐FDA‐approved therapy may contribute to adverse outcomes, including complications that could necessitate surgical interventions.^16^ Our results advocate for a more streamlined regulatory process that prioritizes the pediatric population to ensure timely access to safe and effective treatments. There is currently a lack of prospective safety and pharmacokinetic data in pediatrics which limits optimized and safe dosing for these vulnerable age groups. There are, however, multiple reviews and cohort studies that have been conducted in review of biologics, small molecules, and combination therapy which have summarized their safety profiles.^24^, ^25^, ^26^, ^27^, ^28^ These reviews are limited by small cohort sizes and lack of long‐term data, but they demonstrate generally favorable safety profiles. Initiatives to include pediatric populations in clinical trials could bridge this gap and enhance access to safe and effective treatment options.^29^, ^30^

Limitations of our study include its retrospective design and reliance on registry data, which may not capture all relevant clinical variables affecting treatment decisions by physicians. Conversely, there is an advantage to utilizing this data set as it was prospectively collected over the course of clinical management. Additionally, the observational nature of this study does not allow us to establish causality between demographic factors and medication exposure. The ICN Registry collects only limited data on safety and adverse events, so this study does not address any potential risks of off‐label medication use. Future studies should consider longitudinal designs and incorporate qualitative methods to better understand the decision‐making processes of healthcare providers regarding the use of non‐FDA‐approved medications in pediatric IBD.

## 5 CONCLUSIONS

Our study highlights a significant issue within pediatric IBD management: the reliance on medications not approved for this demographic. A considerable proportion of pediatric patients diagnosed with IBD are receiving treatments that have not been approved by the FDA for pediatric use and this promotes discussion on dosing safety in this population. The pharmacokinetics of many of these medications are not fully understood, may vary in children, and thus could result in inappropriate dosing without proper investigation in this cohort.^31^, ^32^ This study's analyses suggest that younger patients suffer from more aggressive disease and need quicker access to approved medications to prevent disease progression, preventable complications, growth failure, and disturbances to prepubertal development. This underscores an urgent need for regulatory agencies to prioritize the approval of IBD medications for the pediatric population to diminish risks for long‐term and life‐threatening complications. Addressing this with increased speed to FDA approval is essential to ensure that young IBD patients have access to necessary treatments.

## CONFLICT OF INTEREST STATEMENT

The authors declare no conflicts of interest.

## Supporting information

ICN FDA Supplemental Table 1.

ICN FDA Supplemental Authors.

ICN FDA Figure 1.docx.

ICN FDA Figure 2.docx.

supmat.
