# Supplementary material for: A significant number of pediatric inflammatory bowel disease patients are exposed to a medication not approved by the Food and Drug Administration for pediatric use
Source: J Pediatr Gastroenterol Nutr. 2025 Aug 25;81(5):1208–15. doi: 10.1002/jpn3.70200 (PMC12580454; doi:10.1002/jpn3.70200)
Supplement: Supplementary file 1 — ICN FDA Supplemental Table 1. [file JPN3-81-1208-s002.docx]

| Year of Diagnosis (total number of patients diagnosed) | Number of patients exposed to non-FDA approved medication (%) |
| --- | --- |
| 2007 (n=351) | 43 (12.2) |
| 2008 (n=401) | 49 (12.2) |
| 2009 (n=524) | 70 (13.4) |
| 2010 (n=598) | 95 (15.9) |
| 2011 (n=803) | 142 (17.7) |
| 2012 (n=882) | 155 (17.6) |
| 2013 (n=998) | 160 (16.0) |
| 2014 (n=1104) | 182 (16.5) |
| 2015 (n=1223) | 200 (16.4) |
| 2016 (n=1307) | 273 (20.9) |
| 2017 (n=1269) | 275 (21.7) |
| 2018 (n=1320) | 297 (22.5) |
| 2019 (n=1315) | 286 (21.8) |
| 2020 (n=1203) | 234 (19.5) |
| 2021 (n=1079) | 184 (17.0) |
| 2022 (n=261) | 31 (11.9) |
