# Supplementary material for: A significant number of pediatric inflammatory bowel disease patients are exposed to a medication not approved by the Food and Drug Administration for pediatric use
Source: J Pediatr Gastroenterol Nutr. 2025 Aug 25;81(5):1208–15. doi: 10.1002/jpn3.70200 (PMC12580454; doi:10.1002/jpn3.70200)
Supplement: Supplementary file 2 — ICN FDA Supplemental Authors. [file JPN3-81-1208-s003.docx]

The following authors are from the ImproveCareNow Pediatric IBD Learning Health System and should be listed in PubMed as authors. All of these authors participated in the conceptualization of the design of the work and/or data acquisition, drafting/review of the work, and final approval of the manuscript.

Jennifer Strople Ann & Robert H. Lurie Children’s Hospital of Chicago

Jeffrey A Bornstein Arnold Palmer Hospital for Children

Tiffany Linville Atrium Health Levine Children’s Hospital

Salman S Salman Bon Secour/ St Mary’s Hospital

Leslie Higuchi Boston Children’s Hospital

Rashmi Patil Children’s Hospital at Erlanger

Gitit Tomer Children’s Hospital at Montefiore, Albert Einstein College of Medicine

Teena Sebastian Children’s National Hospital

Mark E Kusek Children’s Nebraska

Ian Kang University of Illinois College of Medicine Peoria

Rana Ammoury Children’s Hospital of The King’s Daughters

Kathryn Clarkston Children’s Mercy Hospital

Kenneth E Grant CHOC Children’s Hospital of Orange County

Peter A Margolis Cincinnati Children’s Hospital Medical Center

Kaitlin Whaley Cincinnati Children’s Hospital Medical Center

Tina Morhardt Dartmouth Health Children’s

Kelly C Sandberg Dayton Children’s Hospital, Boonshoft School of Medicine, Wright State University

Dyer Heintz Dell Children’s Medical Center

John L Lyles Duke University Health System

Harohalli Shashidhar Elliot Pediatric Gastroenterology, Elliot Hospital Manchester

Barbara J Niklinska-Schirtz Emory University School of Medicine

Dana MH Dykes GI Care for Kids

Keith J Benkov Icahn School of Medicine at Mount Sinai

Jillian S Sullivan Larner College of Medicine at the University of Vermont

Melissa Sheiko Legacy Health, Randall Children’s Northwest Gastroenterology

Noah JF Hoffman MaineHealth Barbara Bush Children’s Hospital

Colleen LeBlanc Manning Family Children’s

Jess L Kaplan Mass General for Children, Harvard Medical School

Michael C Stephens Mayo Clinic

Jose Cabrera Medical College of Wisconsin

Carmine Suppa Medical University of South Carolina

Roy Nattiv MemorialCare Miller Children’s & Women’s Hospital, Long Beach

Jeremy Adler Mott Children’s Hospital, University of Michigan

Brendan Boyle Nationwide Children’s Hospital

J Fernando del Rosario Nemour’s Children’s Health Delaware

Jill Dorsey Nemour’s Children’s Health Jacksonville

Alisa J Muniz Crim Nicklaus Children’s Hospital

Tuvia A Marciano NYU Langone Pediatric Gastroenterology Associates- Lake Success

Brian G Morris Ochsner Children’s Hospital

Jeanne Tung Oklahoma University Medical Center

Howard I Baron Pediatric Gastroenterology and Nutrition Associates

Marc E Schaefer Penn State Hershey Children’s Hospital

Brad A Pasternak Phoenix Children’s Hospital

Yuliya Rekhtman Prisma Health - Midlands

Liz Dancel Prisma Health- Upstate

Jeannie S Huang Rady Children’s Hospital - San Diego

David L Suskind Seattle Children’s Hospital

Helen M Pappa SSM Cardinal Glennon Children’s

Meryl Perlman St. Louis Children’s Hospital – Washington University

Rachel Bensen Stanford University School of Medicine

Jeffrey A Morganstern Stony Brook Children’s Hospital

Trusha Patel The Children’s Hospital of Philadelphia

Daphne Say UC Davis Children’s Hospital

Sabina Ali UCSF Benioff Children’s Hospital Oakland

Denise D Young UH/Rainbow Babies & Children’s Hospital, Case Western University School of Medicine

Traci W Jester University of Alabama at Birmingham/ Children’s of AL

Jennifer L Dotson University of Arkansas for Medical Sciences

Brad D Constant University of Colorado School of Medicine

Genie Beasley University of Florida

Dawn R Ebach University of Iowa

Vikram J Christian University of Minnesota

Razan Alkhouri University of New Mexico

Ajay S Gulati University of North Carolina at Chapel Hill

Craig A McKinney University of Virginia

Daniel M O’Connell University of Wisconsin School of Medicine and Public Health

Whitney M Sunseri UPMC Children’s Hospital of Pittsburgh

Galen S Hartman Upstate Golisano Children’s Hospital, SUNY Upstate Medical University

Bhaskar Gurram UT Southwestern Medical Center

Diana C Riera Vanderbilt University School of Medicine

Amy J Guido West Virginia University School of Medicine
