# Supplementary figures and images for: A significant number of pediatric inflammatory bowel disease patients are exposed to a medication not approved by the Food and Drug Administration for pediatric use
Source: J Pediatr Gastroenterol Nutr. 2025 Aug 25;81(5):1208–15. doi: 10.1002/jpn3.70200 (PMC12580454; doi:10.1002/jpn3.70200)

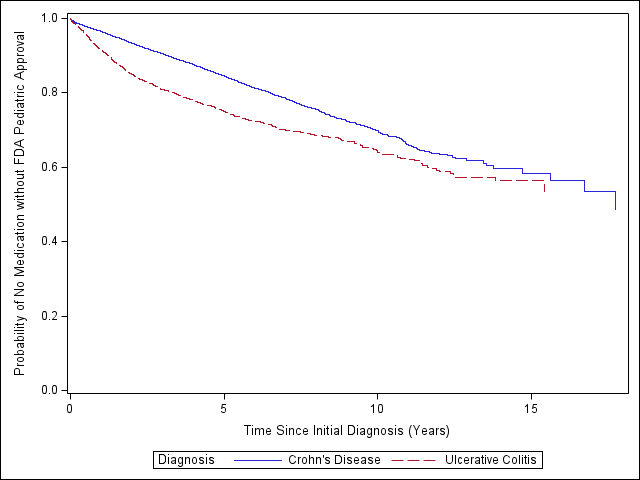

Supplement: Supplementary file 3 — ICN FDA Figure 1.docx. [file JPN3-81-1208-s004.docx]

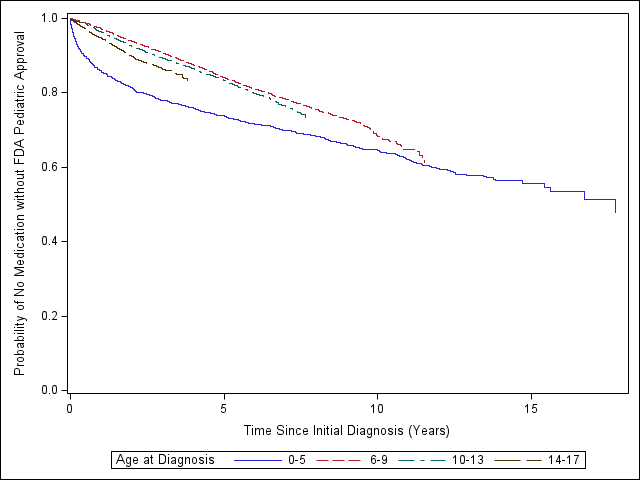

Supplement: Supplementary file 4 — ICN FDA Figure 2.docx. [file JPN3-81-1208-s001.docx]
