# Supplementary material for: A significant number of pediatric inflammatory bowel disease patients are exposed to a medication not approved by the Food and Drug Administration for pediatric use
Source: J Pediatr Gastroenterol Nutr. 2025 Aug 25;81(5):1208–15. doi: 10.1002/jpn3.70200 (PMC12580454; doi:10.1002/jpn3.70200)
Supplement: Supplementary file 5 — supmat. [file JPN3-81-1208-s005.docx]

**Supplemental Table Legend**

Supplemental Table 1. Patients Exposed to Any Medication without FDA Pediatric Approval by Year of Diagnosis (2007-2022)

**Supplemental Figure Legends**

Supplemental Figure 1: Kaplan-Meier Analysis Demonstrating Different Rates of Exposure to Medications without FDA Pediatric Approval by Inflammatory Bowel Disease Diagnosis (p▒<▒0.001).

Supplemental Figure 2: Kaplan-Meier Analysis Demonstrating Different Rates of Exposure to Medications without FDA Pediatric Approval by Age at Diagnosis.
